# Controlling mechanical properties of bio-inspired hydrogels by modulating nano-scale, inter-polymeric junctions

**DOI:** 10.3762/bjnano.5.101

**Published:** 2014-06-23

**Authors:** Seonki Hong, Hyukjin Lee, Haeshin Lee

**Affiliations:** 1 Department of Chemistry, Center for Nature-inspired Technology in KI NanoCentury, Korea Advanced Institute of Science and Technology (KAIST), 291, University Rd, Daejeon 305-701, South Korea; 2 College of Pharmacy, Graduate School of Pharmaceutical Sciences, Ewha Womans University, Seoul 120–750, South Korea

**Keywords:** catechols, hydrogels, poly(ethylene glycol)s, quinone tanning

## Abstract

Quinone tanning is a well-characterized biochemical process found in invertebrates, which produce diverse materials from extremely hard tissues to soft water-resistant adhesives. Herein, we report new types of catecholamine PEG derivatives, PEG-NH-catechols that can utilize an expanded spectrum of catecholamine chemistry. The PEGs enable simultaneous participation of amine and catechol in quinone tanning crosslinking. The intermolecular reaction between PEG-NH-catechols forms a dramatic nano-scale junction resulting in enhancement of gelation kinetics and mechanical properties of PEG hydrogels compared to results obtained by using PEGs in the absence of amine groups. Therefore, the study provides new insight into designing new crosslinking chemistry for controlling nano-scale chemical reactions that can broaden unique properties of bulk hydrogels.

## Introduction

**Figure 1:**
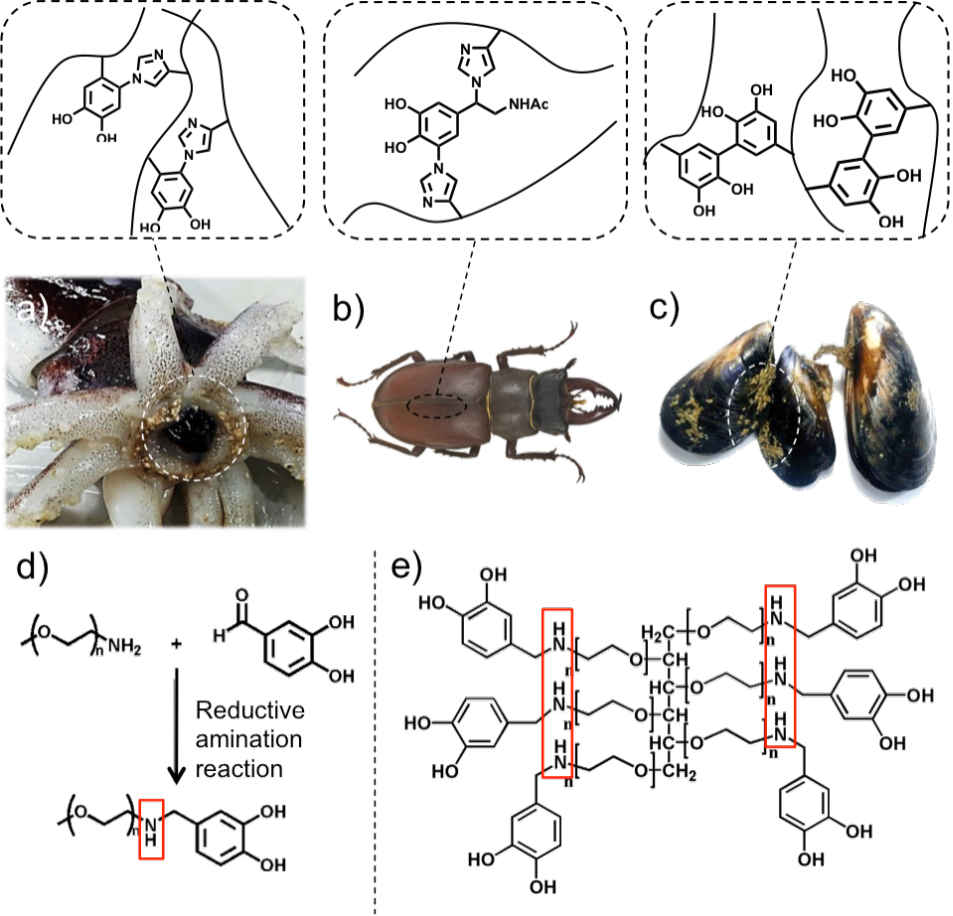
Biomaterials formed by quinone tanning processes found in (a) squid beaks, (b) insect cuticles, and (c) mussel adhesives. Representative chemical reactions were shown for each biomaterials (a,b,c top). Synthetic PEG derivatives that can mimic the natural catecholamine-involved quinone tanning due to the presence of secondary amine*:* (d) mPEG-NH-catechol for a model reaction, and (e) 6Arm-PEG-NH-catechol for hydrogels.

Water-resistant adhesives secreted by marine mussels, stiff cuticles synthesized by insects, and sharp beaks found in squids appear to be drastically different biomaterials (Figure 1a–c) [1–6]. Not only their mechanical properties, but also their biological functions are distinct: The adhesives anchor mussels in place for survival and colonization, the cuticles securely protect insects from predators, pathogens, and environmental stresses, and the beaks act as a non-mineralized knife for capturing prey.

However, despite such differences in biological function, the molecular basis for the formation of the beaks, the cuticles, and the adhesives is similar. The process is called quinone tanning, which is defined by chemical crosslinking of proteins by a variety of reactive quinones. For mussel adhesives, DOPAquinone is formed by oxidation of a catecholic amino acid, 3,4-dihydroxy-L-phenylalanine (DOPA). Subsequently, DOPAquinone rapidly reacts with basic amino acids, such as lysine and/or histidine, forming covalent adducts of DOPA-DOPA, lysyl-DOPA, and/or histeinyl-DOPA [7–9]. For insect cuticles, the quinone tanning (i.e., sclerotization) occurs via crosslinking of cuticular proteins in which primary amines, secondary amines, and phenols from the proteins react with *N*-acetylcatecholamines [9–11]. For squid beaks, the reaction between the imidazole of histidine and DOPAquinone is the primary mechanism for mechanical hardening of the beaks (Figure 1a–c) [6].

The quinone tanning process has been a useful method to create chemically functionalized interfaces regardless of the chemistry of materials. Recently, we and other research groups have reported novel strategies for the functionalization of virtually any material surfaces by using synthetic catecholamine polymers such as poly(dopamine) [12], poly(norepinephrine) [13], and poly(ethylenimine)-catechol [14]. The surfaces modified by those catecholamines exhibited a variety of functionalities such as protein-immobilization [15], facilitating cell adhesion [16], attenuating in vivo toxicity [17], initiating bio-mineralization [18], graphene nano-composites [19], and bio-inspired adhesives [20–21]. In addition to the interface science and engineering, methods to prepare bulk materials such as poly(ethylene glycol) (PEG) and pluronic hydrogels have been reported [22–26]. However, most previous work utilized catechol–catechol crosslinking by using catechol end-functionalized polymers, which limits the control of important variables in hydrogels such as gelation kinetics and mechanical properties.

Herein, we report new types of PEG derivatives (linear mPEG-NH-catechol and branched 6Arm-PEG-NH-catechol) that offer an expanded spectrum of catecholamine chemistry. In the PEG derivatives, catechol and secondary amine coexist that can effectively mimic the chemical process of catecholamine-involved quinone tanning. The hydrogels produced by catecholamine crosslinking using 6Arm-PEG-NH-catechol exhibited enhanced mechanical properties and rapid gelation compared to the hydrogel prepared by PEGs that can only use catechol-catechol crosslinking. This study demonstrates that the chemical configuration by inserting both secondary amine and catechol expands the properties of PEG hydrogels, which can provide new insight into designing hydrogels prepared by other polymers using quinone tanning chemistry.

## Results and Discussion

### Quinone tanning reactions of catecholamine PEGs

Preparation of linear (5 kDa) and 6Arm-PEG-NH-catechol (15 kDa) was performed by a simple one-step reductive amination between 3,4-dihydroxybenzaldehyde (DHBA) and linear or 6Arm-PEG-NH_2_ (Figure 1d and Figure 1e). Primary amines are difficult to be chemically tethered to PEG-catechol, because the typical reaction (i.e., EDC coupling) is the formation of an amide bond between the primary amine and the carboxyl group. Instead, we could easily generate secondary amines by using aldehyde chemistry, in other words reductive amination reaction, for the PEG-catechol to contain secondary amine groups. Secondary amines are also well-known to be reactive with catechol, which has been found in natural organisms [6]. The product was purified by dialysis (MWCO = 3 kDa for linear PEG and MWCO = 10 kDa for 6Arm-PEG) for two days and was subsequently lyophilized. White powders were obtained for both PEG derivatives. An absolute negative result from the ninhydrin test indicated that all amine groups of linear and 6Arm-PEGs had reacted with the catechol derivatives. Control polymers with the absence of secondary amines, linear and 6Arm-PEG-catechols, were prepared by an amide bond forming reaction using the BOP/HOBt/DIPEA coupling with 3,4-dihydroxyhydrocinnamic acid (DHCA) and linear or 6Arm-PEG-NH_2_.

To investigate the effect of secondary amines on the results of quinone tanning, both linear PEG derivatives, mPEG-NH-catechol and mPEG-catechol, underwent the same crosslinking reactions in phosphate buffered saline (pH 8.0 with 0.3 mM NaIO_4_) (Supporting Information File 1, Figure S6). Quantitative analysis of the crosslinked PEG products was performed by gel permeation chromatography (GPC). GPC results showed that the peak that appeared at 18 min of elution time is unreacted monomer (Standard data of unreacted monomer: Supporting Information File 1, Figure S5), and the other peaks eluted at around 17 min or earlier indicate multimers resulting from the quinone tanning reactions. The peak intensities of the multimers and the monomer were varied depending on the concentration of NaIO_4_, which initiated the quinone tanning reaction. It was found that maximum intensity of the multimer peaks was obtained at 1.5 equiv NaIO_4_ to catechols for both mPEG-NH-catechol (Supporting Information File 1, Figure S6, left) and mPEG-catechol (Supporting Information File 1, Figure S6, right). The entire range of NaIO_4_ added to the reaction mixtures was 0.25–2 equiv to catechols. Amounts of NaIO_4_ higher than 1.5 equiv no longer affected the results of the crosslinking. In fact, the intensity of the multimer peak for mPEG-NH-catechol was decreased, when 2 equiv of NaIO_4_ were used. One equivalent of NaIO_4_ to catechol is enough, but in practice a slight excess of NaIO_4_ was found to be necessary for maximal crosslinking.

### Quantitative analysis of crosslinked PEG products with GPC

**Figure 2:**
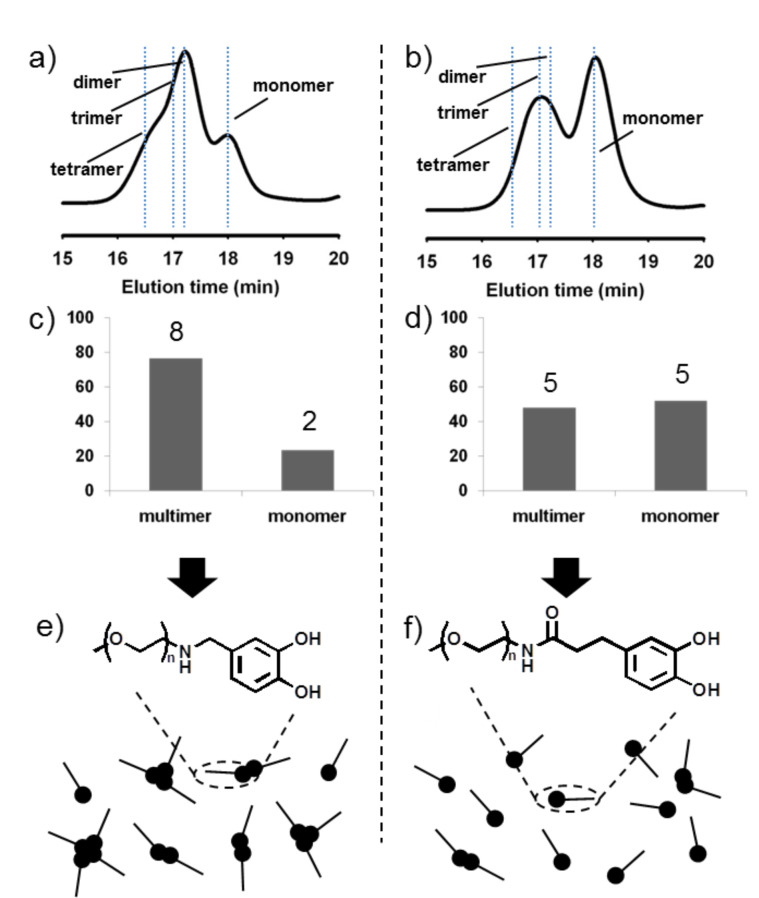
Comparative analysis of crosslinking products of mPEG-NH-catechol and mPEG-catechol. (a,b) GPC data obtained from crosslinking at a stoichiometric ratio of NaIO_4_:catechol = 1.5:1. The dot lines indicate each elution time of PEG standards with molecular weight of 5, 10, 15, and 20 kDa. The quantitative multimeric and monomeric ratio of crosslinked mPEG-NH-catechol and mPEG-catechol were shown in (c) and (d). (e,f) Schematic results comparing nanoscale junctions formed by (e) mPEG-NH-catechol and (f) mPEG-catechol.

One notable result was that the relative amounts and the molecular species of the multimers formed by quinone tanning reactions exhibited large differences between mPEG-NH-catechol and mPEG-catechol. For mPEG-NH-catechol, multimers were a major component (Figure 2a) but became a minor molecular species for mPEG-catechol. The monomer was dominant in the mPEG-catechol reaction mixture (Figure 2b).

The molecular weight analysis of the GPC products showed that the multimer peak consisted of dimeric, trimeric, and tetrameric PEGs, composing up to about 80 percent of the total product in the mPEG-NH-catechol reaction mixture (Figure 2c). However, for mPEG-catechol, the GPC analysis showed that only dimeric and trimeric PEGs was formed. No indication of tetramers was found in the reaction mixture, and a large amount of unreacted monomer, about 50 percent, remained (Figure 2d). The lines in Figure 2a and Figure 2b indicate the elution time of standard PEG compounds with known molecular weight and configuration. Linear mPEG-NH_2_ 5 kDa (18.0 min elution time), 10 kDa (17.2 min), 4Arm-PEG-NH_2_ 20 kDa (16.5 min) were used as standards (Supporting Information File 1, Figure S5). These results demonstrated that the quinone tanning reaction involved with amine and catechol simultaneously was more efficient than the reaction engaged only with catechol (Figure 2e and 2f). This result strongly suggests that one can control physicochemical properties of a wide variety of PEG-containing biomaterials by designing effective conjugation chemistry*.* We chose PEG hydrogels as an example.

### Effect of the amine group in PEG gelation I: mechanical properties of hydrogels

**Figure 3:**
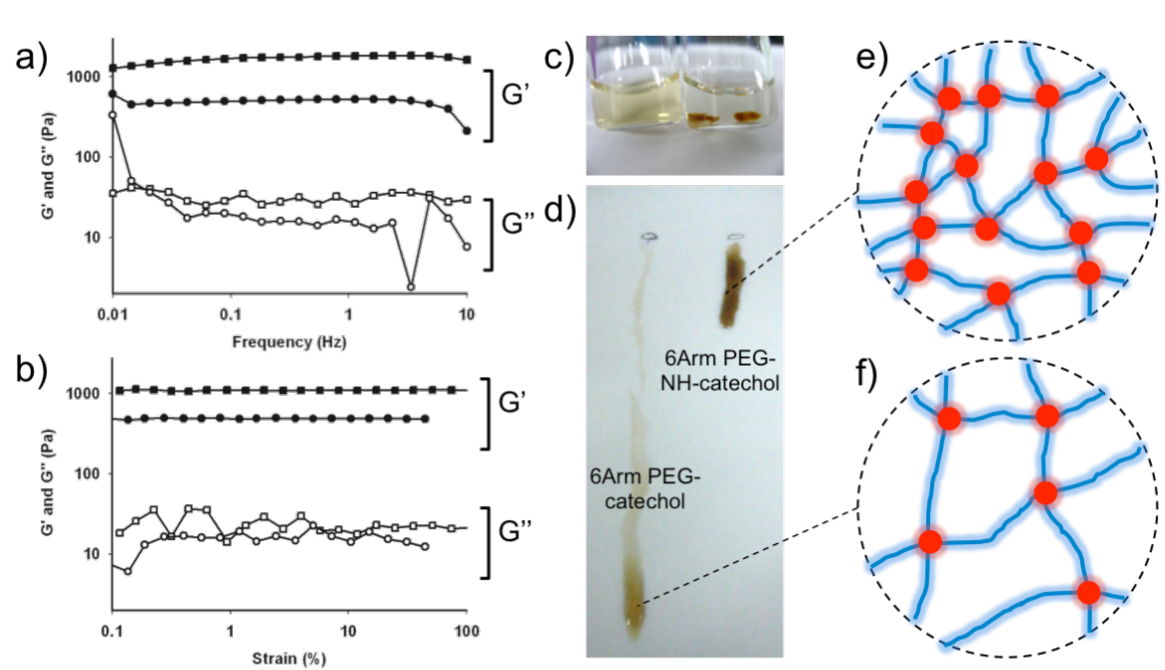
Rheological analysis data of quinone tanning inspired crosslinking hydrogels (a) frequency sweep and (b) strain sweep of 6Arm-PEG-NH-catechol (square) and 6Arm-PEG-catechol (circle). (n = 3 each) (c) Rapid quinone tanning of 6Arm-PEG-NH-catechol (right) in NaIO_4_ (100 mM in DDW). A visible tanning reaction was not observed in 6Arm-PEG-catechol (left). (d) Measurement of gelation kinetics by dropping solutions of 6Arm-PEG-NH-catechol (right) and 6Arm-PEG-catechol (left) on a PTFE surface (45 deg slope). (e and f) Schematic results showing nano-scale junctions formed by (e) 6Arm-PEG-NH-catechol and (f) 6Arm-PEG-catechol.

To explore the effect of the amine group in PEG gelation, we prepared 6Arm-PEG-NH-catechol and 6Arm-PEG-catechol (control). Both were dissolved in PBS pH 8.0 with a final concentration of 3% (w/v). It is expected that the hydrogel formed by amine-catechol involved tanning (6Arm-PEG-NH-catechol) may exhibit faster gelation kinetics and stronger mechanical properties than the one formed by a catechol–catechol tanning (6Arm-PEG-catechol). We measured the mechanical modulus of the hydrogels by a rheometer. Frequency (0.01 to 10 Hz, Figure 3a) and strain (1 to 90%, Figure 3b) sweeps were performed for three times each on both 6Arm-PEG-NH-catechol and 6Arm-PEG-catechol hydrogels, after allowing time for complete gelation (10 min). The elastic modulus, G’, and loss modulus, G”, were found to be independent over a wide range of frequencies and strains, demonstrating that the gelation was successfully completed within 10 min. The elastic modulus of the hydrogel made of 6Arm-PEG-NH-catechol was about 1,000 Pa, but the G’ of the hydrogel prepared by 6Arm-PEG-catechol was low, as expected (about 500 Pa). The two-times increase in the elastic modulus indicates that amine-catechol quinone tanning is more chemically efficient process compared to catechol–catechol tanning. Therefore, we understand that the amine–catechol tanning process has been chosen in nature to produce stiff biomaterials for the various invertebrates shown in Figure 1.

### Effect of the amine group in PEG gelation II: differences in gelation kinetics

In rheology, gelation point is defined by the intersection of the elastic modulus (G’) and the loss modulus (G”). We tried to measure the point by a rheometer, but it was found that gelation occurred within a minute in both hydrogels (6Arm-PEG-NH-catechol and 6Arm-PEG-catechol), preventing a direct measurement of the gelation time in a rheometer. There was, however, a difference in gelation kinetics: dropping the solution of 6Arm-PEG-NH-catechol (3%, w/v) into a NaIO_4_ solution (100 mM in DDW) immediately formed dark brown gel-like aggregates (Figure 3c, right bottle). In contrast, such rapid aggregates were not detected when dropping the 6Arm-PEG-catechol solution was dropped into the same solution (Figure 3c, left bottle). The PEG solution was dispersed in the NaIO_4_ solution. This clear indication of differences in the gelation time led us to design a new, simple method for measuring this parameter. A poly(tetrafluoroethylene) (PTFE) surface was set up at a 45° slope angle, and drops of 6Arm-PEG-NH-catechol (Figure 3d, right) and 6Arm-PEG-catechol (Figure 3d, left) solutions were applied to observe the gelation time. The PEG solution flowed in the liquid phase but stopped in the gel phase. Immediate gelation of 6Arm-PEG-NH-catechol was observed within 2 s, but it took about 12 s for the 6Arm-PEG-catechol solution to become a hydrogel. This result, combined with the rheology data, indicates that amine–catechol tanning is an efficient process that is suitable for controlling a large number of chemical junctions in nanobiomaterials (Figure 3e and Figure 3f). Because the reaction occurs in aqueous conditions, many invertebrates utilize this process to produce a variety of biomaterials for their use in nature. The effective tanning process of catechol and amine demonstrated herein suggests a general approach for creating novel catecholaminergic derivatives of biopolymers such as alginate, hyaluronic acid, chitosan, dextran, and other synthetic or proteineous materials for a variety of applications.

**Figure 4:**
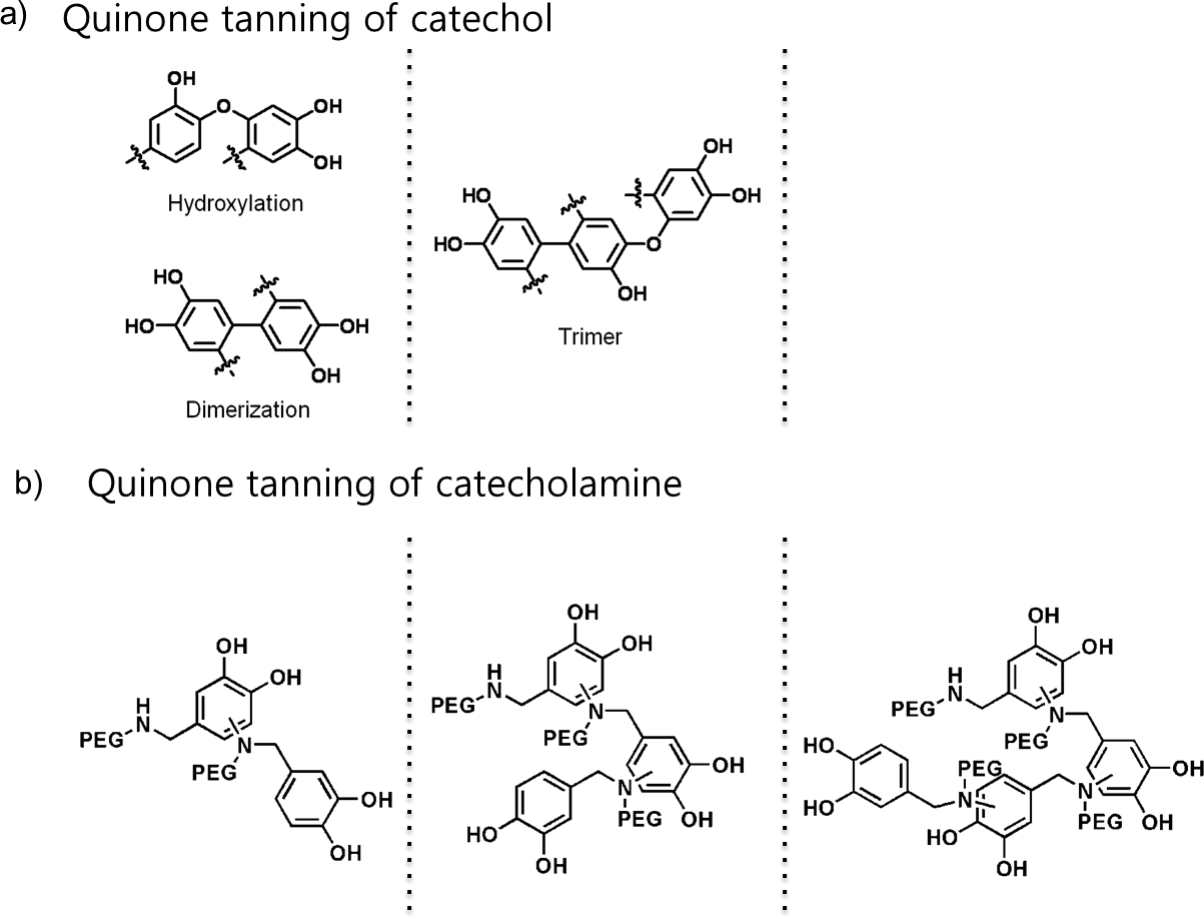
Proposed chemical structures of crosslinked products by quinone tanning reactions. (a) Catechol quinone tanning and (b) catecholamine quinone tanning. In catecholamine tanning, amine-involved crosslinking shown in (b) is more favorable than catechol tanning pathway shown in (a).

Quinone, an oxidized form of catechol, is reactive to nucleophiles such as hydroxyl, amine, and quinone, which typically undergo the 1,4-Michael addition reactions [27]. Considering the molecular structures of PEGs studied herein, three quinone tanning reactions are possible: (i) catechol–catechol formation through C–C coupling between phenyl rings [27–28], (ii) C–O coupling at a C-4 position by the reaction between deprotonated hydroxyl anion and quinone [27,29], and (iii) C–N coupling at the same position by the secondary amine and quinone [7–11,27]. For PEG-catechol, the C–C and C–O couplings are the only possible quinone tanning mechanisms to form hydrogels (Figure 4a). However, for PEG-NH-catechol, another scenario of C–N coupling is added to the two previous tanning reactions (catechol–catechol and C–O couplings of PEG-catechol) (Figure 4b). It is generally accepted that the nucleophilicity of amines is higher than that of a hydroxyl group. Thus, nature utilizes the amine-involved quinone tanning reactions for the formation of stiff insect cuticles [7–11,27] by a reaction between imidazole (side-chain of histidine) and catechol. So far, the majority of research was focused on catechol–catechol crosslinking [25,30–31], accidentally ignoring the importance of catecholamine quinone tanning. Thus, the results demonstrated herein can be a useful toolkit to further control physicochemical properties of biomaterials.

## Conclusion

In summary, we demonstrated that the particular quinone tanning process simultaneously involved with catechol and amine was effective in crosslinking. The linear type of polymer mPEG-NH-catechol showed superior crosslinking result compared to mPEG-catechol, the polymer crosslinked via catechol-catechol tanning. Similar to the difference in crosslinking efficiency in linear PEG experiments, hydrogels formed by multi-armed PEGs showed that the hydrogel utilizing catechol–amine tanning, 6Arm-PEG-NH-catechol, showed fast in gelation kinetics (about 2 s) and strong in mechanical properties (G' > 1,000 Pa) compared to the hydrogel produced by 6Arm-PEG-catechol (about 12 s and about 500 Pa).

## Experimental

### Materials

6Arm-PEG-amine (PEG-(NH_2_)_4_, *M*_w_ = 15,000 Da) and methoxy-PEG-amine (mPEG-NH_2_, *M*_w_ = 5,000 Da) were purchased from SunBio, Inc. (Walnut Creek, CA). Sodium periodate (NaIO_4_), sodium cyanoborohydride (NaBH_3_CN), *N*,*N*-diisopropylethylamine (DIPEA), 3,4-dihydroxyhydrocinnamic acid (DHCA) and 3,4-dihydroxybenzaldehyde (DHBA) were acquired from Sigma-Aldrich (St. Louis, MO). 1-Hydroxybenzotriazole (HOBt) hydrate was purchased from Peptides International (Louisville, KY) and benzotriazole-1-yl-oxy-tris-(dimethylamino)-phosphonium hexafluorophosphate (BOP) was acquired from Novabiochem (Germany).

### Synthesis of catechol-conjugated PEG (1)

Synthesis of 6Arm-PEG-catechol and mPEG-catechol: 6Arm-PEG-amine (1 g, 0.067 mmol) was dissolved in 10 mL of NMP at 60 °C for 10 min. DHCA (0.8 mmol), BOP (0.8 mmol), HOBt (0.8 mmol) and DIPEA (0.8 mmol) were dissolved in 5 mL of NMP in a separate vial. The PEG and DHCA solutions were mixed and reacted at room temperature for 3–6 h. The reaction solution was purified by dialysis at acidic condition for 2 d and then lyophilized. (Dialysis conditions: 10 mL of polymer solution was added to 3 L of DDW for 3 h and the dialysate was exchanged for four times. MWCO = 5,000.) The content of catechol was confirmed by UV–vis spectroscopy at 280 nm. The UV–vis intensity of catechol modified polymer was calculated to the contents of catechol by using the standard curve made by the known concentration of dopamine solution versus the UV–vis intensity, demonstrating that all terminal amine groups were conjugated with DHCA. The catechol contents of catechol-modified PEG were double checked by the ninhydrin test. The ninhydrin test was performed by mixing 20 μL of catechol-modified PEG (1 mg mL^−1^ in DDW) with 20 μL of 2% ninhydrin reagent solution, followed by heating at 100 °C for 3 min. The heated solution was diluted to 700 µL of DDW and then the primary amine and secondary amine was confirmed by UV–vis spectrometer at 470 nm and 440 nm. ^1^H NMR (300 MHz, CDCl_3_, δ): 6.71–6.69 (m, 2H, C_6_HH_2_(OH)_2_-), 6.52–6.49 (dd, 1H, C_6_H_2_H(OH)_2_-), 3.79–3.33 (m, PEO), 2.81–2.76 (t, 2H, C_6_H_3_(OH)_2_-CH_2_-), 2.49–2.44 (t, 2H, CH_2_-C(O)NH-). mPEG-catechol was prepared by using the same procedure described above with the amount of reagents used are the followings: mPEG-amine (200 mg, 0.04 mmol), DHCA (0.048 mmol), BOP (0.048 mmol), HOBt (0.048 mmol) and DIPEA (0.048 mmol). The Purity of synthetic PEGs was determined by ^1^H NMR and GPC (Supporting Information File 1, Figure S2 and S4).

### Synthesis of catechol-conjugated PEG (2)

6Arm-PEG-amine (200 mg, 0.013 mmol) was solved in 2 mL of NMP at 60 °C for 10 min. DHBA (33 mg, 0.24 mmol) in 1 mL of NMP was added to the PEG solution and stirred at room temperature for 1 h. NaBH_3_CN (38 mg, 0.6 mmol) in 100 µL of NMP was added to that solution and reacted at room temperature for overnight. The reaction solution was successively purified by dialysis at acidic condition for 2 d and then lyophilized. The content of catechol was confirmed by ultraviolet–visible spectroscopy at 280 nm, demonstrating that all terminal amine groups were conjugated with DHBA. (The dialysis condition and catechol content assay were the same as in the synthesis of 6Arm-PEG-catechol and mPEG-catechol.) ^1^H NMR (300 MHz, CDCl_3_, δ) 7.19 (s, 1H, C_6_H_2_H(OH)_2_-), 6.81–6.88 (m, 2H, C_6_HH_2_-(OH)_2_-), 4.09–4.05 (m, 2H, -NH-CH_2_-C_6_H_3_(OH)_2_-), 3.95–3.59 (m, PEO), 3.40–3.36 (t, 2H, PEO-CH_2_-NH-). mPEG-NH-catechol was prepared by using the same procedure described above with the amount of reagents used being as follows: mPEG-amine (200 mg, 0.04 mmol), DHBA (17 mg, 0.12 mmol), NaBH_3_CN (38 mg, 0.6 mmol). The purity of synthetic PEGs was determined by ^1^H NMR and GPC (Supporting Information File 1, Figure S1 and S3).

### Quinone tanning reactions of catecholamine PEGs

Quinone tanning reactions of mPEG-NH-catechol and mPEG-catechol were performed by the addition of sodium periodate (NaIO_4_) to PEG solutions (2 mg mL^−1^ in 10 mM phosphate buffered saline, pH 8.5), and the resulting molecular weight of the crosslinked PEGs was determined by gel permeation chromatography (GPC). Two GPC columns (OHpak SB-806M HQ and SB-804 HQ, Shodex®, Munich, Germany) were connected in series and equilibrated with phosphate buffered saline (10 mM, pH 4.0). The detector for the PEG standard was a reflective index detector (Shodex, RI-71), and a UV–vis spectrometer (Hewlett Packard HP 8453 spectrophotometer, 190 nm to 1100 nm, integration time 0.5 sec, interval 1 nm, Deuterium lamp for UV and tungsten lamp for vis) was used for detecting the catechol modified polymers. The columns were characterized with various PEG standards: mPEG-amine (linear, 5 kDa), mPEG-amine (linear, 10 kDa), 4Arm-PEG-amine (star shaped, 20 kDa). The elution time of the PEG standards were 18 min for mPEG-NH_2_ (linear, 5 kDa), 17.2 min for mPEG-amine (linear, 10 kDa), 16.5 min for 4Arm-PEG-NH_2_ (star shaped, 20 kDa). Our GPC column approximately separated the 100 Da molecular weight after 30 min elution time, calculated by using a standard curve of 130 kDa, 30 kDa, 20 kDa, and 5 kDa molecular weight hyaluronic acid and PEGs. (Supporting Information File 1, Figure S5) The quinone tanning reaction was optimized by varying the amount of sodium periodate (0.1 to 2 equiv relative to mole of catechol). It was confirmed in our previous report that the chemical pathway of catechol crosslinking is exactly the same whether NaIO_4_ is used or not, and NaIO_4_ can effectively control the kinetics of catecholamine crosslinking without any additional byproducts [32].

### Formation of PEG hydrogels

To form PEG hydrogels, 1.5 equiv of NaIO_4_ (moles of catechol basis) was added to 3% PEG solution (30 mg mL^−1^ in phosphate-buffered saline, pH 8.0), and the subsequently determined gelation time through vial inversion method was typically 10–20 s. For a naccurate determination of the gelation time, we used PTFE plate setup with 45 degree slope and droplets of PEG solutions (10 µL) allowed to flow until the solutions stopped when became hydrogels.

### Oscillatory rheometry

Oscillatory rheometry by using a rotating rheometer (Bohlin Advanced Rheometer with a parallel 20 mm plate, Malvern Instruments, UK) was used to determine the mechanical properties of the PEG hydrogels. Strain sweep was performed at 1 Hz and frequency sweep was carried out at a strain of 10% after 10 min from the gel formed. To perform rheology studies of the modified-PEG hydrogels, 1.5 equiv of NaIO_4_ (moles of catechol basis) was added to 3% PEG solution (30 mg mL^−1^ in phosphate-buffered saline, pH 8.0) in 24 well-plate as a mold for rheology sample. The formed hydrogel was a cylinder shape with diameter of 16 mm and height of 1 mm. The time dependent rheology test showed that the sol–gel transition was finished within 1 min (data not shown). In order to test the mechanical properties of the hydrogels in a stable condition, we carried out the frequency and strain sweep test 10 min after the gel formed.

## Supporting Information

File 1 Further experimental data.
The further experimental data describes ^1^H NMR and GPC data for the purity of synthetic products, mPEG-catechol, mPEG-NH-catechol, 6Arm-PEG-catechol, and 6Arm-PEG-NH-catechol.
