# Supplementary material for: Controlling mechanical properties of bio-inspired hydrogels by modulating nano-scale, inter-polymeric junctions
Source: Beilstein J Nanotechnol. 2014 Jun 23;5:887–94. doi: 10.3762/bjnano.5.101 (PMC4077523; doi:10.3762/bjnano.5.101)
Supplement: File 1 — Further experimental data. The further experimental data describes 1H NMR and GPC data for the purity of synthetic products, mPEG-catechol, mPEG-NH-catechol, 6Arm-PEG-catechol, and 6Arm-PEG-NH-catechol. [file Beilstein_J_Nanotechnol-05-887-s001.pdf]

Supporting Information

for

Controlling mechanical properties of bio-inspired hydrogels by modulating nano-scale, inter-polymeric junctions

Seonki Hong[1], Hyukjin Lee[2] and Haeshin Lee*[1]

Address: [1]Department of Chemistry, Center for Nature-inspired Technology in KI NanoCentury, Korea Advanced Institute of Science and Technology (KAIST), 291, University Rd, Daejeon 305-701, South Korea and [2]College of Pharmacy, Graduate School of Pharmaceutical Sciences, Ewha Womans University, Seoul 120–750, South Korea

Email: Haeshin Lee – haeshin@kaist.ac.kr

* Corresponding author

Further experimental data

^{1}H NMR data of 6Arm-PEG-NH-catechol and 6Arm-PEG-catechol

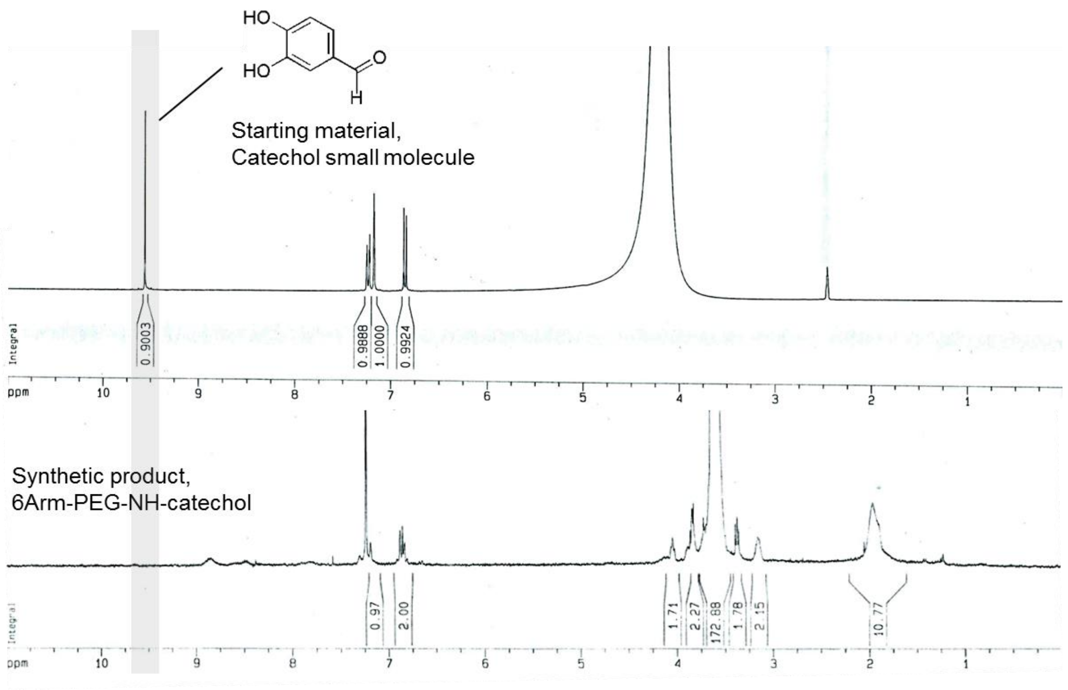

Figure S1. Purity confirmation of synthesized 6Arm-PEG-NH-catechol polymer analyzed by ^{1}H NMR. Peaks at 9–10 ppm indicating aldehyde in starting material (up), were successfully disappeared after bound to PEGs (down).

6Arm-PEG-NH-catechol ^{1}H NMR (300 MHz, $CDCl_3$, δ)

7.19 (s, 1H, $C_6H_2H(OH)_2$-), 6.81-6.88 (m, 2H, C_6HH_2-$(OH)_2$-), 4.09-4.05 (m, 2H, -NH-CH_2-$C_6H_3(OH)_2$-), 3.95-3.59 (m, PEO), 3.40-3.36 (t, 2H, PEO-CH_2-NH-)

3,4-dihydroxybenzaldehyde (DHBA, 300 MHz, DMSO-*d*$_6$, δ)

9.56 (s, 1H, CHO), 7.26-7.22 (dd, 1H, $C_6H_2H(OH)_2$-CHO), 7.18 (d, 1H, $C_6H_2H(OH)_2$-CHO), 6.87-6.84 (d, 1H, $C_6H_2H(OH)_2$-CHO)

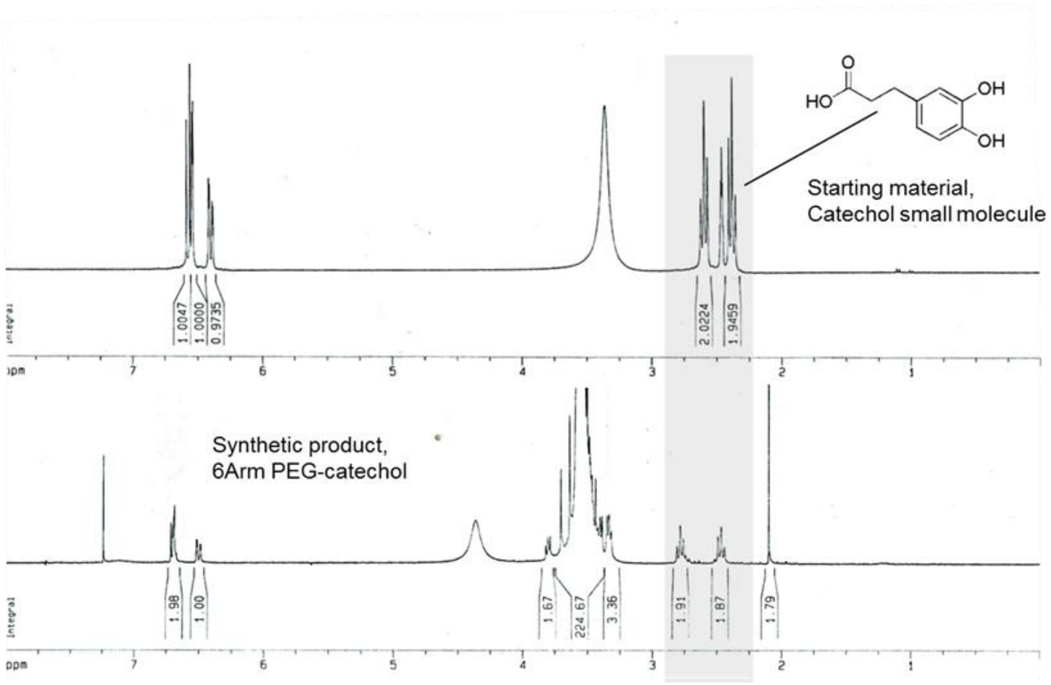

Figure S2. Purity confirmation of synthesized 6Arm-PEG-catechol polymer analyzed by ^{1}H NMR. Peaks at 2–3 ppm, -CH_2-CH_2- in starting material (up), were successfully shifted with 0.2 ppm after bound to PEGs (down).

6Arm-PEG-catechol ^{1}H NMR (300 MHz, $CDCl_3$, δ)

6.71-6.69 (m, 2H, $C_6HH_2(OH)_2$-), 6.52-6.49 (dd, 1H, $C_6H_2H(OH)_2$-), 3.79-3.33 (m, PEO), 2.81-2.76 (t, 2H, C6H3(OH)2-CH2-), 2.49-2.44 (t, 2H, CH2-C(O)NH-)

3,4-dihydroxyhydrocinnamic acid (DHCA, 300 MHz, DMSO-d_6, δ)

6.59-6.56 (d, 1H, C_6H2H$(OH)_2$-), 6.55-6.54 (d, 1H, $C_6H_2H(OH)_2$-), 6.42-6.39 (dd, 1H, $C_6H_2H(OH)_2$-), 2.62-2.57 (t, 2H, $C_6H_3(OH)_2$-CH_2-), 2.41-2.36 (t, 2H, -CH_2-COOH)

GPC data of mPEG-NH-catechol and mPEG-catechol

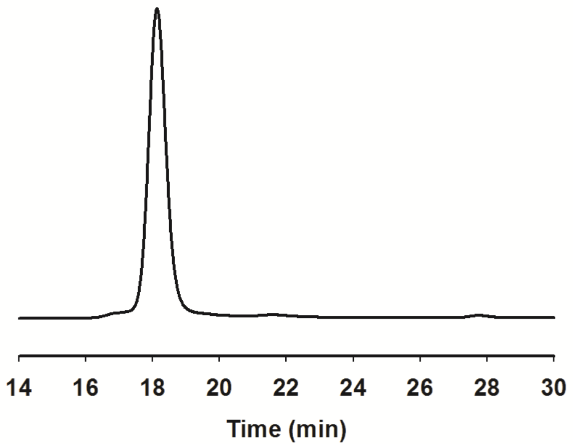

Figure S3. GPC data of synthesized mPEG-NH-catechol. There were no peaks after 18 min, elution time of synthesized mPEG-NH-catechol product.

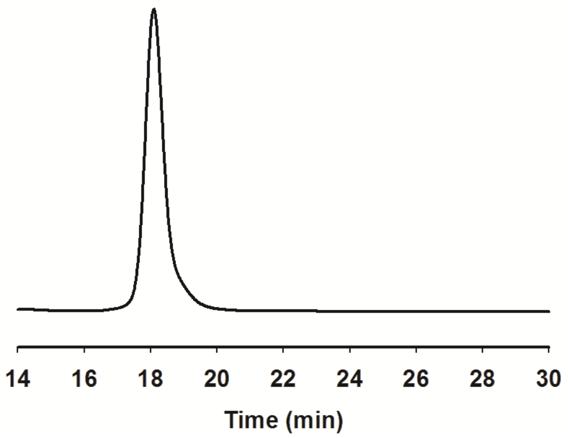

Figure S4. GPC data of synthesized mPEG-catechol. There were no peaks after 18 min, elution time of synthesized mPEG-catechol product.

Calibration curve of our GPC system

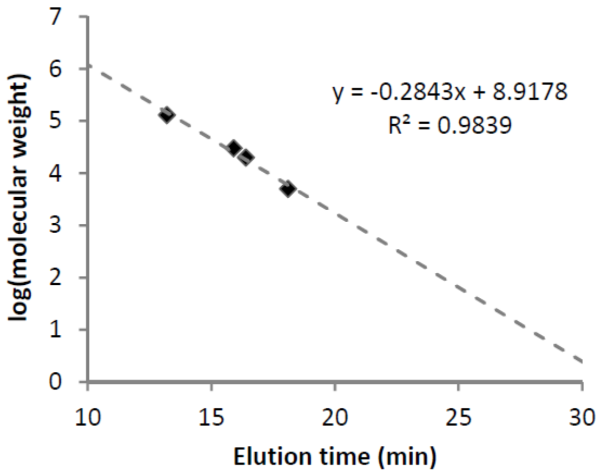

Figure S5. Calibration curve of GPC system. Each point means 130 kDa, 30 kDa, 20 kDa, and 5 kDa molecular weight Hyaluronic acid and PEGs. 30 min corresponds to the molecular weight under 100 Da. This means above GPC data of mPEG-NH-catechol and mPEG-catechol have no impurities, molecular weight under 5 kDa to 100 Da.

Quinone tanning reactions of catecholic linear PEG derivatives.

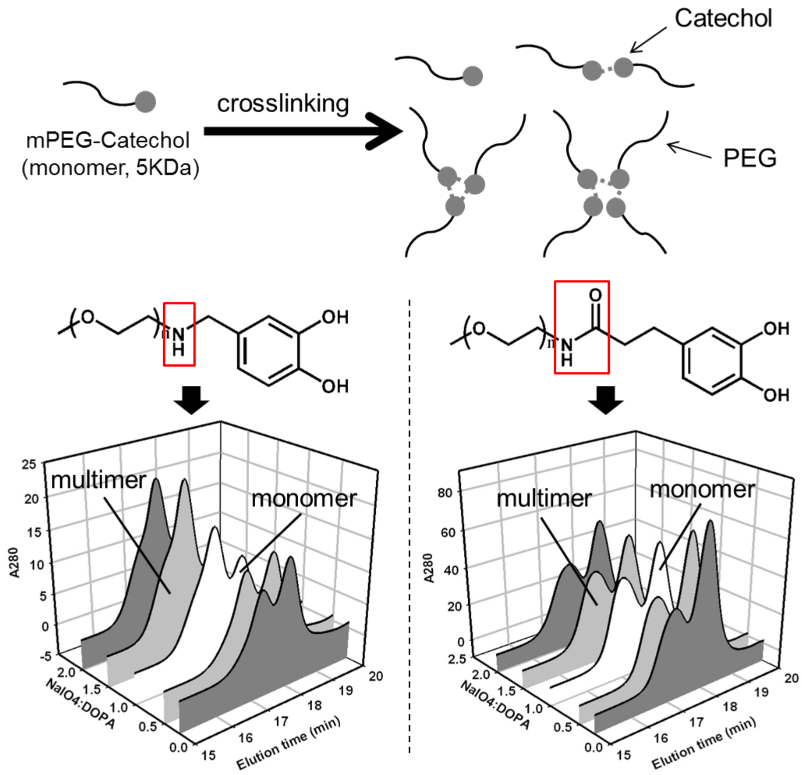

Figure S6. A schematic description of quinone tanning (i.e. crosslinking) of catecholic PEGs (Chemical structure: mPEG-NH-catechol (left) and mPEG-catechol (right)) Quinone tanned products of mPEG-NH-catechol and mPEG-catechol as a function of a stoichiometric ratio of $NaIO_4$: catechol (0.25 : 1 – 2 : 1) was analyzed by gel permeation chromatography.
